# Reliability, repeatability, and reproducibility of pulmonary transit time assessment by contrast enhanced echocardiography

**DOI:** 10.1186/s12947-015-0044-1

**Published:** 2016-01-05

**Authors:** Ingeborg H. F. Herold, Salvatore Saporito, R. Arthur Bouwman, Patrick Houthuizen, Hans C. van Assen, Massimo Mischi, Hendrikus H. M. Korsten

**Affiliations:** 1 Department of Anesthesiology and Intensive-Care, Catharina Hospital Eindhoven, Michelangelolaan 2, 5623 EJ Eindhoven, The Netherlands; 2 Department of Electrical Engineering, Signal Processing Systems, Eindhoven University of Technology, De Zaale, 5612 AZ Eindhoven, The Netherlands; 3 Department of Cardiology, Catharina Hospital Eindhoven, Michelangelolaan 2, 5623 EJ Eindhoven, The Netherlands

**Keywords:** Contrast enhanced ultrasound, Echocardiography, Indicator dilution technique, Intra-class correlation, Mean transit time, Pulmonary transit time, Reliability

## Abstract

**Background:**

The aim of this study is to investigate the inter and intra-rater reliability, repeatability, and reproducibility of pulmonary transit time (PTT) measurement in patients using contrast enhanced ultrasound (CEUS), as an indirect measure of preload and left ventricular function.

**Methods:**

Mean transit times (MTT) were measured by drawing a region of interest (ROI) in right and left cardiac ventricle in the CEUS loops. Acoustic intensity dilution curves were obtained from the ROIs. MTTs were calculated by applying model-based fitting on the dilution curves. PTT was calculated as the difference of the MTTs. Eight raters with different levels of experience measured the PTT (time moment 1) and repeated the measurement within a week (time moment 2). Reliability and agreement were assessed using intra-class correlations (ICC) and Bland-Altman analysis. Repeatability was tested by estimating the variance of means (ANOVA) of three injections in each patient at different doses. Reproducibility was tested by the ICC of the two time moments.

**Results:**

Fifteen patients with heart failure were included. The mean PTT was 11.8 ± 3.1 s at time moment 1 and 11.7 ± 2.9 s at time moment 2. The inter-rater reliability for PTT was excellent (ICC = 0.94). The intra-rater reliability per rater was between 0.81–0.99. Bland-Altman analysis revealed a bias of 0.10 s within the rater groups. Reproducibility for PTT showed an ICC = 0.94 between the two time moments. ANOVA showed no significant difference between the means of the three different doses F = 0.048 (*P* = 0.95). The mean and standard deviation for PTT estimates at three different doses was 11.6 ± 3.3 s.

**Conclusions:**

PTT estimation using CEUS shows a high inter- and intra-rater reliability, repeatability at three different doses, and reproducibility by ROI drawing. This makes the minimally invasive PTT measurement using contrast echocardiography ready for clinical evaluation in patients with heart failure and for preload estimation.

**Electronic supplementary material:**

The online version of this article (doi:10.1186/s12947-015-0044-1) contains supplementary material, which is available to authorized users.

## Background

Pulmonary blood volume quantification by transpulmonary dilution analysis is an essential part of the hemodynamic evaluation to guide fluid management in anesthesia and intensive care practice. Recently, contrast-enhanced ultrasound (CEUS) has been proposed as a minimally-invasive, alternative method for pulmonary transit time (PTT) estimation [1–3]. This technique uses transthoracic echocardiography (TTE) to visualize the transcardiac passage of an ultrasound contrast-agent (UCA) bolus injected in a peripheral vein. Indicator dilution curves (IDCs) are then derived from the acoustic backscatter of the UCA bolus in the four heart chambers. The mean transit time (MTT) of these acoustic IDCs can be estimated by different methods. The most frequently used methods in clinical practice are based on assessment of the “peaks” of the IDCs or “frame counting” of the appearance of the first bubbles in the heart chambers [3–5]. We estimate the MTT by model fitting using the local density random walk (LDRW) model, which takes into account the Brownian motion of the bolus contrast in the blood stream through the pulmonary vessels and heart chambers [1, 2, 6, 7].

In previous studies, we demonstrated that volume estimation by CEUS, resulting from the multiplication of the flow by the PTT (i.e. the difference between the MTTs of the left atrium and the right ventricle (RV)), showed excellent agreement with the actual volumes, both in-vitro and in-vivo [2, 6]. Moreover, it showed even better accuracy than transpulmonary thermodilution volume estimation [1, 8]. However, the reliability of PTTs derived with CEUS and the LDRW in-vivo has not been established. Therefore, in this study we investigated the reliability and reproducibility of the assessment of PTT with CEUS using the LDRW model, in patients referred for cardiac resynchronization therapy. We also investigated the effect of different UCA doses on the PTT measurement. In addition, to evaluate the complexity of the PTT assessment by means of CEUS-recording analysis, PTT was also estimated by non-physicians. If also non-physicians can obtain reliable measurements, this would imply a fast learning curve, favoring the method adoption in clinical practice. Therefore, our second objective was to evaluate the reliability and agreement between PTT measurements obtained by physicians and non-physicians.

## Methods

### Patients

As per local hospital protocol, all patients referred for cardiac resynchronization therapy underwent extensive echocardiographic evaluation including contrast enhanced ejection fraction measurements. This patient population scheduled for contrast enhanced TTE to assess ejection fraction and eligibility were included for this observational study. In general, these were patients with symptomatic heart failure, a decreased ejection fraction, and QRS-widening by more than 120 ms. Patients were excluded in case of atrial fibrillation, an acute coronary syndrome within the past three months, a known allergy to sulphur-hexafluoride, or a poor acoustic window (impossibility to visualize an apical 4-chamber view). The Institutional Review Board of the Catharina Hospital Eindhoven approved the study, and written informed consent for use of echocardiography data for scientific purposes was obtained from all subjects.

### Measurement protocol

**Fig. 1:**
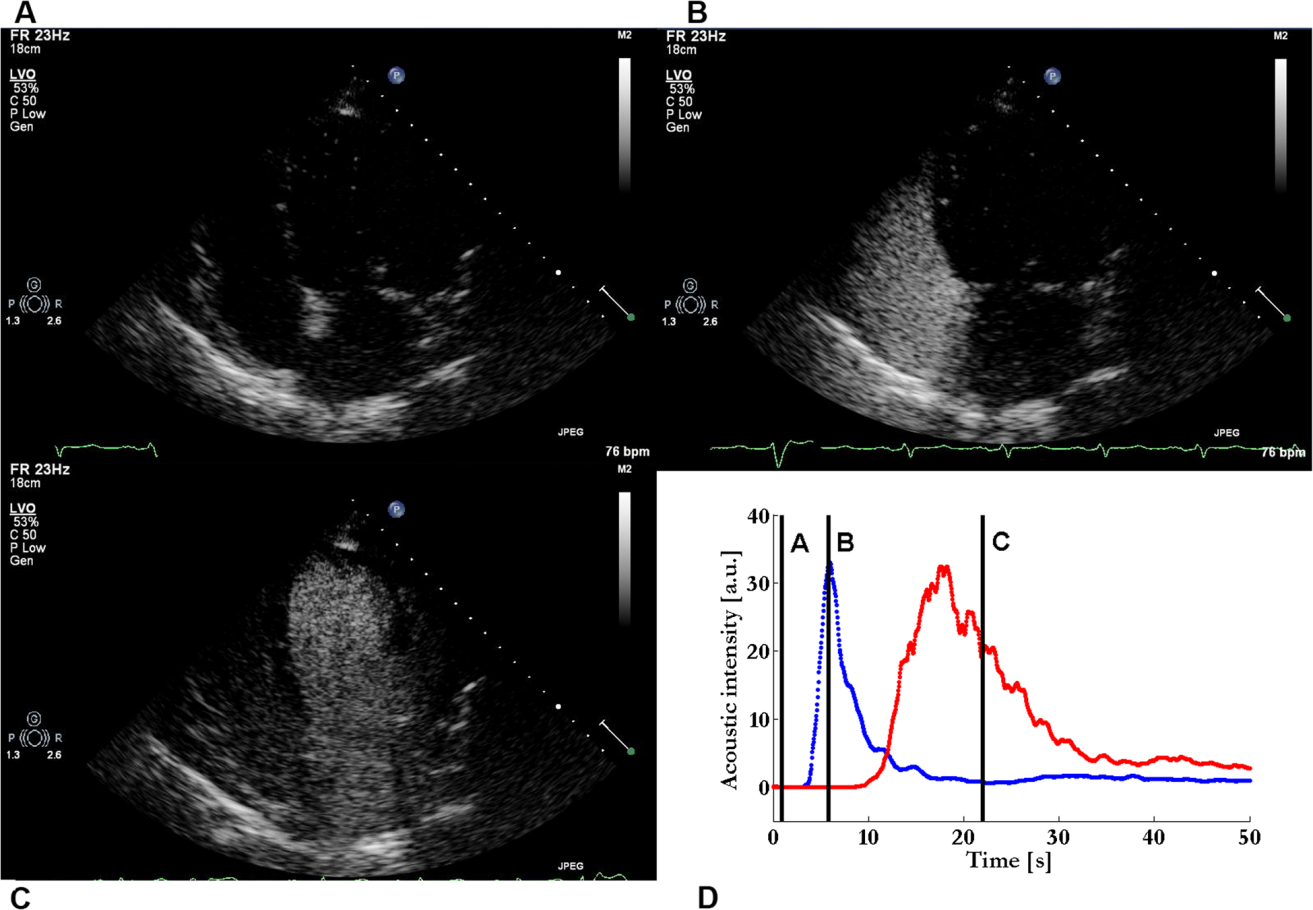
The passage of a bolus of SonoVue® through the right atrium and ventricle (panel **b**) and left atrium and ventricle (panel **c**). ROIs are drawn in the right and left ventricle (not shown, see Additional file 1). The acoustic intensities according to panel **a**, **b**, and **c** are expressed in panel **d**. The acoustic dilution curves of the right (blue indicator dilution curve (IDC)) and left ventricle (red IDC) are fitted according to the local density random walk model and the mean transit times (vertical black lines) are then calculated

Patients referred for a left ventricle (LV) dyssynchrony evaluation received a standard of care CEUS echocardiography according to our hospital protocol (Fig. 1). In all patients, an 18-gauge catheter was inserted in a peripheral vein of the fore-arm and the patient was positioned in left lateral position. The UCA was administered according to our hospital CEUS protocol. All contrast-enhanced TTE imaging was performed by an experienced imaging-cardiologist (PH) using an iE33 ultrasound scanner equipped with a S5-1 transducer (Philips Healthcare, Andover, MA, USA). Four chamber apical views were obtained using harmonic imaging at 1.3–2.6 MHz, a low mechanical index (MI) of 0.19 to reduce microbubble destruction, a frame rate of 23 Hz, and a dynamic range of 50 dB with linear post-processing.

SonoVue® (Bracco SpA, Milan, Italy) consisting of microbubbles with a SF_6_ gas enclosed in a phospholipids monolayer shell was used as UCA. These microbubbles, with an average size of 3 to 9 μm, pass the pulmonary circulation and can be visualized in both RV and LV [9]. After a peripheral bolus injection, the passage of microbubbles through the right and left heart was visualized from a four chamber apical view without breath-hold by selecting the proper probe angle, to minimize movement artifacts. We diluted 1 ml SonoVue® in saline (1:400, 1:200, and 1:100) and injected a bolus of 10 ml of the solution. Three different CEUS loops were recorded in consistent consecutive order. The injections were performed in a reproducible way as a manual instantaneous bolus and each dose was administered as soon as the recirculating microbubbles disappeared from the left ventricle. These are doses lower than those used for left ventricular opacification [10], but give good opacification of the ventricles while providing an approximately linear relationship between the UCA concentration and the measured acoustic intensity; the latter is a prerequisite for application of the indicator dilution theory [1, 2]. Reliability was tested by eight raters: four physicians who were all experienced with echocardiography but with different levels of experience with measuring MTT (one rater was experienced (IH), one rater had some experience (HK), and two had experience in endocardial border tracing (PH, JD)), and four technicians from the Eindhoven University of Technology of whom three were inexperienced with echocardiography and MTT (HA, GW, AG) and one was an expert in the field (MM). All raters received a detailed guide for drawing ROIs in both RV and LV at the two time moments. Each rater received the same fifteen anonymized ultrasound apical four chamber view loops following the injection of a SonoVue® in saline bolus at a dilution of 1:200 (Fig. 1). The raters were instructed to draw regions of interest (ROIs) independently within the endocardial borders of the RV and LV throughout the cardiac cycle; no specified ROI surface was predefined (Additional file 1). ROI position was fixed over all the frames. Movement of borders or valve structures within the ROI will create artifacts in the IDC. Therefore, the instructions emphasized that the ROIs should be kept within the endocardial border during the entire cardiac cycle. To this end, the freeform splines ROIs were drawn using QLab® 8 software (Philips, Healthcare, Andover, MA, USA). The acoustic intensity over time in the ROIs was expressed as an IDC. To test the reproducibility of the measurement itself, the raters repeated the drawings in the same dataset within a week interval; these time points are referred to as time moment 1 and 2. The raters were blinded for their previous results and to the measurement outcome; also the order of the patients in the dataset was changed between time moment 1 and 2. The extracted data were saved as Excel files and were afterwards analyzed using a custom software to fit the local density random walk (LDRW) model to the measured IDCs; the method was implemented in MATLAB® 2009b (The Mathworks, Natick, MA, USA) [11]. The interdose repeatability was tested by one rater (IH) who drew ROIs in all three different echo loops at the three different doses for the fifteen patients. Thereby, ROI size was measured and the difference in MTT in the fifteen cine loops at three different doses.

The IDCs were fitted by the LDRW model by an independent researcher (GW). The fitting was performed automatically as described by Mischi et al. [11]. The analysis of the acoustic IDC provides parameters related to the convection and dispersion of the injected SonoVue® bolus including the MTT [2, 12]. The difference in MTT (ΔMTT) between LV and RV, presenting the PTT, was derived by subtraction [7].

### Statistical analysis

The analysis and rendering of this observational study are in line with the guideline for reporting reliability and agreement studies (GRRAS) [13]. The intra-class correlation for random effects models based on repeated-measures ANOVA was used to evaluate intra-rater and inter-rater reliability [14]. These were analyzed for the different time moments 1 and 2 and the different classes: physicians versus technicians. The level of agreement between the PTT measured by physicians and technicians was determined with Bland-Altman analysis to estimate the feasibility of CEUS using MedCalc Statistical Software version 14.8.1 (MedCalc Software bvba, Ostend, Belgium) [15]. The distributions of the MTTs of both ventricles and the PTTs were tested by the Shapiro-Wilk test. The MTTs and PTTs are presented as mean and standard deviation (for normal distributions) or median and interquartile ranges (for non-normal distributions). Coefficients of variation were measured for the PTT and computed among the rater groups. The repeatability of the three different doses and different loops was tested using univariate ANOVA analysis. The ROI size was tested for its distribution as described above for PTT. Statistical analyses were performed using IBM SPSS statistics for Windows version 22.0 (IBM^©^, Armonk, NY, USA).

## Results

Fifteen patients (12 men and 3 women) were enrolled in the study. Mean age was 67 ± 7 years with an ejection fraction of 31 ± 11 % (Table 1). The transit times at any time moment had a normal distribution according to the Shapiro-Wilk test. The mean MTT of the RV was 8.5 ± 3.1 s and 8.7 ± 3.1 s at time moment 1 and 2, respectively. For the LV, these values were 20.4 ± 5.6 s and 20.3 ± 5.5 s, respectively. The mean PTT was 11.8 ± 3.1 s at time moment 1 and 11.7 ± 2.9 s at time moment 2.

**Table 1:** Demographic characteristics

	Mean ± SD (*n* = 15)	Min – max (*n* = 15)
Gender (n male/women)	12/3	
Age (yr)	67 ± 7	58–78
Weight (kg)	81 ± 15	51–109
Height (cm)	176 ± 9	158–188
BMI	26 ± 4	19–37
BSA	2.0 ± 0.2	1.5–2.3
NYHA classification n (%)		
Class II	7 (47)	
Class III	5 (33)	
Class IV	3 (20)	
Echocardiography		
LVEF (%)	31 ± 11	17–53
RV dysfunction		
TAPSE < 16 mm (n)	4/15	
Pulmonary hypertension		
TR velocity > 2.8 m/s (n)	2/15	
Electrocardiogram		
Heart rate	72 ± 17	50–110
QRS duration (ms)	151 ± 27	118–194
IVMD (ms) in 13 patients	42 ± 14	20–60
SPWMD (ms) in 11 patients	152 ± 70	10–240
Comorbidities n (%)		
Hypertension	5 (33)	
Coronary artery disease	11 (73)	
Congestive heart failure	14 (93)	
COPD	6 (40)	
Diabetes mellitus	3 (20)	
Medication n (%)		
Beta-blocker	12 (80)	
ACE inhibitors	11 (73)	
AT II blockers	2 (13)	
Loop diuretics	12 (80)	
K-sparing agents	6 (40)	
Statins	14 (93)	
Laboratory		
Creatinine (μmol/L)	105 ± 28	31–160
NT-proBNP (pmol/L)	162 ± 111	36–440

*SD* standard deviation, *BMI* body mass index, *BSA* body surface area, *NYHA* New York Heart Association, *LVEF* left ventricular ejection fraction, *RV* right ventricle, *TAPSE* tricuspid annular plane systolic excursion, *TR* tricuspid regurgitation, *IVMD* interventricular mechanical delay, *SPWMD* septal to posterior wall motion delay, *COPD* chronic obstructive pulmonary disease, *ACE* angiotensin-converting enzyme, *AT II* angiotensine-II-receptor antagonist, *K-sparing* potassium sparing, *NT-proBNP* N-terminal pro-B-type natriuretic peptide

**Fig. 2:**
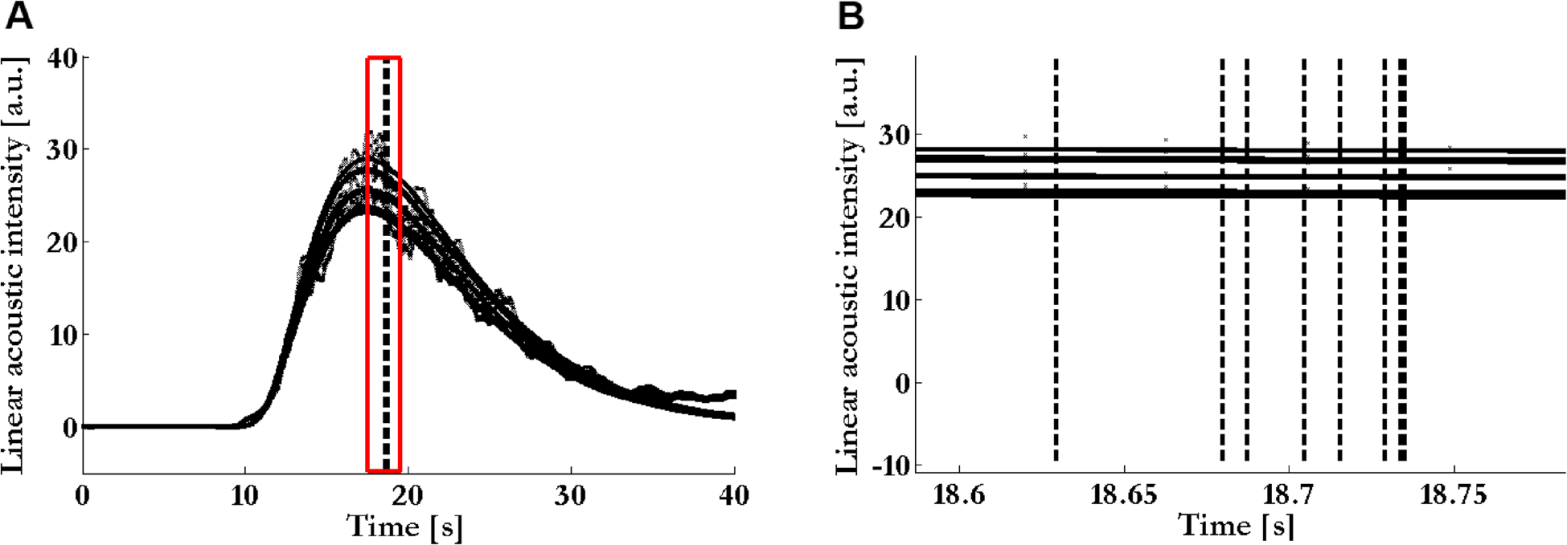
The fitted curves by eight raters of one patient’s left ventricle indicator dilution curve (IDC) (panel **a**). The difference in mean transit times of these fitted IDCs among the eight raters in one patient (panel **b**)

**Fig. 3:**
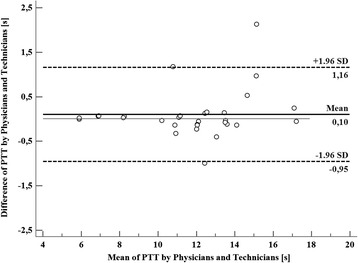
Bland-Altman analysis. The X-axis represents the mean of the average pulmonary transit time (PTT) in the two time moments measured by physicians and technicians. The Y-axis represents the difference of the average PTTs in the two time moments between the physicians and technicians. The solid line is the mean difference (bias); dotted lines are limits of agreement [bias ± (1.96 SD)]

The MTT assessments made at the two different time moments per rater showed a high reliability with intra-class correlation coefficient (ICC) for the PTT equal to 0.94 (95 % CI, 0.90–0.97). For the MTTs of the RV and LV, the ICC was 0.98 (95 % CI, 0.97–0.99) and 0.99 (95 % CI, 0.98–0.99), respectively (Table 2 and Fig. 2). In Fig. 2, the peak of the IDCs of the different ROIs, drawn by the raters, is visualized and its effect on the MTT of the LV evidenced. In this figure, it is shown that the difference in LV MTT is very low among the raters. The coefficient of variation was lowest for the LV (1.21 %) and highest for the PTT (3.30 %) (Table 2). Reproducibility for the measurements between the two time moments performed by all raters demonstrated an ICC of 0.99 (95 % CI, 0.98–0.99), 0.99 (95 % CI, 0.98–0.99), and 0.94 (95 % CI, 0.92–0.94) for the RV MTT, LV MTT, and PTT, respectively (Table 3). Reproducibility between the two time moments for the PTT per rater showed an ICC between 0.81 and 0.99. The ICC was 0.99 for all technicians; for the physicians it varied between 0.81 and 0.99 (Table 4). Bland-Altman analysis revealed a mean difference of 0.10 (±0.54) s between the physicians and technicians. The 95 % limits of agreement ranged from – 0.95 s to 1.16 s (Fig. 3).

**Table 2:** Inter-rater reliability of the mean transit times measured in the right and left ventricle and of the pulmonary transit time of the two time moments between the eight raters. The coefficient of variation is expressed as a percentage

	ICC (95 % CI)	Coefficient of variation (± SD) %
Right ventricle mean transit time	0.98 (0.97–0.99)	2.61 (3.00)
Left ventricle mean transit time	0.99 (0.98–0.99)	1.21 (1.18)
Pulmonary transit time	0.94 (0.90–0.97)	3.30 (3.35)

*ICC* intra-class correlation coefficient, *CI* confidence interval, *SD* standard deviation

**Table 3:** Intra-rater reliability of the mean transit times measured in the right and left ventricle and of the pulmonary transit time by all the raters between the two time moments

	ICC (95 % CI)
Right ventricle mean transit time	0.99 (0.98–0.99)
Left ventricle mean transit time	0.99 (0.98–0.99)
Pulmonary transit time	0.94 (0.92–0.96)

*ICC* intra-class correlation coefficient, *CI* confidence interval

**Table 4:** Intra-rater reliability per rater for the pulmonary transit time (PTT). Raters 1,2,3, and 8 are physicians and raters 4,5,6, and 7 are technicians. The mean PTTs of the fifteen patients are expressed with their standard deviation at time moment 1 and 2, measured by each rater

	ICC (95 % CI)	Mean PTT t1 (± SD)	Mean PTT t2 (± SD)
Rater 1	0.91 (0.76–0.97)	11.86 (3.14)	12.13 (3.14)
Rater 2	0.99 (0.99–1.00)	11.77 (3.00)	11.69 (2.99)
Rater 3	0.92 (0.79–0.97)	11.82 (3.08)	11.49 (3.22)
Rater 4	0.99 (0.99–1.00)	11.64 (2.89)	11.70 (2.96)
Rater 5	0.99 (0.98–1.00)	11.84 (3.09)	11.82 (2.99)
Rater 6	0.99 (0.99–1.00)	11.76 (3.12)	11.71 (3.03)
Rater 7	0.99 (0.98–1.00)	11.65 (2.95)	11.60 (2.93)
Rater 8	0.81 (0.54–0.93)	12.17 (4.15)	11.60 (2.96)

*ICC* intra-class correlation coefficient, *CI* confidence interval, *SD* standard deviation, *t1* time moment 1, *t2* time moment 2

**Fig. 4:**
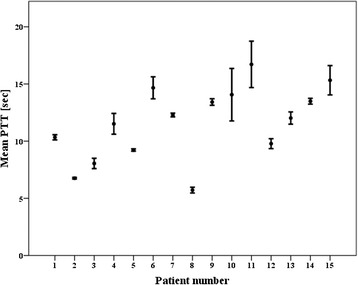
Means of pulmonary transit times in seconds for each patient based on three different SonoVue® concentrations, expressed as bullets and standard deviations as error bars

ANOVA analysis of the three repeated injections showed a mean PTT of 11.4 ± 3.4 s, 11.6 ± 3.4 s, and 11.8 ± 3.2 s at 1:100, 1:200, and 1:400 dilutions of SonoVue® in saline, respectively. The variability amongst the means was F 0.048, which was not significant (*P* = 0.95). The measure of effect for the SonoVue® dose on the PTT accounted for 2 %, η^2^ 0.02. The means and standard deviations of the different PTTs per patient per dose are shown in Fig. 4. The mean PTT and standard deviation for the three different doses was 11.6 ± 3.3 s. The coefficient of variation was 5.3 ± 4.4 %. The ROI sizes were normally distributed and the mean ROI size for the RV was 12 ± 3 mm^2^ and for the LV 25 ± 6 mm^2^. The coefficient of variation of the ROI size based on the three different doses was for the RV 11.3 ± 4.5 % and 9.0 ± 5.3 % for the LV.

## Discussion

This study demonstrated that measurement of PTT derived from IDCs of an UCA bolus injected in a peripheral vein is feasible and highly reliable. The inter- and intra-rater correlations are high, and a very low bias between physicians and technicians was observed. Therefore, the drawing of the ROI in the CEUS recordings is not only reproducible, but also operator independent for measurement of the PTT. According to the high coefficient of variation of the ROI size, the size does not influence the PTT measurement. This makes measurement of the PTT feasible. We also showed that PTT measurement is repeatable, at three different doses in the linear range between concentration and acoustic intensity. The dose did not show any effect on the PTT measurement. The results are in accordance with the volumes measured in an in-vitro model [1]. In this previous study, the volumes were calculated by multiplying the flow through the circuit by the difference in MTT between an inflow and outflow tube [1]. A high ICC of 0.99 (95 % CI, 0.98–1.00) was measured for the three repetitions of an UCA bolus at the same flow and volume of the circuit.

We previously showed that transthoracic and transesophageal CEUS can be used to estimate transit times and pulmonary blood volumes in patients [2, 8]. The values of the PTTs derived with CEUS are in line with cardiopulmonary transit times measured by low-dose contrast-enhanced time-resolved magnetic resonance (MR) angiography, where the magnetic resonance imaging (MRI) signal is measured in the pulmonary artery and ascending aorta [16]. In a patient population similar to ours, with reduced LV function, Shors et al. (2003) reported cardiopulmonary transit times of 11.2 ± 4.0 s in patients with a left ventricular ejection fraction (LVEF) between 21 and 30 % and 9.0 ± 1.7 s for a LVEF between 31 % and 40 % [16]. Our patient population had a mean LVEF of 31 % and the average PTT was 11.8 ± 3.0 s. However, in our cohort two patients had moderate pulmonary hypertension and four patients had moderate right ventricle dysfunction (Table 1), both lead to a prolonged cardiopulmonary circulation times as expressed by PTT [3]. Although in the study of Shors et al., the ROIs were drawn at different regions in the heart and vessels, the transit times are in line with each other. In this study, three-dimensional gradient-echo fast low-angle shots imaging was performed through the pulmonary artery and aorta during inspiration breath-holding [16]. This is different from our study, where the measurements were performed without breath-hold. The advantage of ultrasound is that the temporal resolution of ultrasound is much higher (frame rate 23 Hz) than that of MRI (typically 1 Hz, electrocardiographically triggered on the R-peak depending on the heart rate) [16].

The reliability of our study is in agreement with a recently-published report on the PTT measured in cats using CEUS [17]. The time elapsed to pass the pulmonary circulation by a bolus of SonoVue® was derived by appearance time recording in both the pulmonary artery and left atrium. Veterinarians with different levels of experience evaluated the contrast-enhanced short axis echocardiography loops. Similarly as in our study, no influence of the observers’ experience on PTT measurements was found (median inter-observer variability of 6.8 %) [17]. They concluded that this procedure is simple and robust, and does not need to be limited to experienced operators in echocardiography [17]. Notably, our data suggest the measurement variability to be even lower by the high ICCs (Table 2). This can be explained by the way the individual pulmonary artery or chamber MTTs were estimated. In this study, PTT was estimated by the appearance of contrast behind the pulmonic valve and in the left atrium. Appearance of contrast started or stopped a timer [17]. A similar way to estimate PTT was used by Choi et al. [4]. In this study, investigating transit time as an estimate for cardiac output between the RV and LV, the transit time was 3.2 ± 1.2 s in patients with a mean LVEF of 50 ± 16 % [4]. The transit time was calculated by identification of the first bubble appearance, full opacification, and peak opacification of both ventricles in 27 patients [4]. Incorporating a model like the LDRW, in the MTT assessment, makes it less subjective to human errors. The model-free parameters do not take into account the underlying kinetics of the UCA bolus in the blood stream, like the Brownian motion described by the LDRW model [12, 18]. Though the transit time estimated by two observers at two time moments showed a high intra-observer correlation (1 month interval) of 0.92, the inter-observer correlation was lower (0.79) [4]. This can be explained by the analyses of the UCA passage, by the timing of the first bubble appearance to full opacification of the ventricles; this problem could be solved by using LDRW model fitting, as shown in our results with a higher inter-rater reliability. In the feasibility study of the PTT measurement, an ICC of 0.94 was found in nine patients and two observers using frame counting [3]. This emphasizes the simplicity of this procedure, which by using a model-based method may become more accurate.

The total dosage of SonoVue® used to perform the complete examination according to the protocol was 0.875 mg, which is less than the recommended dose by Bracco. We did not encounter any side effects of SonoVue®. Blood pressure, electrocardiogram, and heart rate were monitored during the examination, and the intravenous access was left in place for 30 min. The chance of side effects has been shown to be low; in a large study with 23,188 abdominal CEUS procedures the overall reporting rate of serious adverse events was 0.0086 % [19]. The chance of any serious allergic reactions has been shown to have a very low incidence (estimated to be 1:10 000) [10].

We used for the analyses three injections of SonoVue® at a total amount of 0.875 mg SonoVue®. Thus, we kept all our doses in the linear range between concentration of SonoVue® and acoustic intensity measured by the ultrasound scanner, avoiding attenuation and shadowing effects [1, 2]. It has been shown that, provided that no shadowing occurs, the effect of the ultrasound scanner settings on the errors in linearization of the video data exported in DICOM files are kept to a minimum when the dynamic range is exceeding approximately 45 dB [20]. Then a wide range of gain values can be used with excellent agreement with those derived from raw radio-frequency data [20]. The percent of error on the MTT measurements, derived from patients with colorectal liver metastasis, at 50 dB and a gain value of 50 dB was 2.8 ± 3.1 % [21]. At lower dynamic range settings, these error percentages were higher [21].

### Study Limitation

Although promising, some limitations should be considered in the interpretation of our results. Sensitivity of the PTT measurement was not addressed in this study. However, several authors reported over a range of cardiac function good correlation with the measured PTT by CEUS. A cut-off point of approximately 4.5 s distinghuished normals from patients with diminished function [3, 4]. In our population, with diminished ejection fractions (± 30 % and dyssynchrony), PTT was 11.2 s. A good differentiation can be appreciated between the different ventricular functions based on PTT-measurements, which is also reported in MRI studies as described above [16, 22].

It is known that intra-cardiac shunts due to for example atrial septum defect or ventricular septum defect will influence the IDC [23]. These shunt characteristics are well known in transpulmonary thermodilution where a thermistor is positioned in the femoral artery. In case of a left-to-right shunt in patients with a ventricular septum defect the tail of the IDC will show an extra humb as the indicator will be recycled from the left to the right atrium [23]. In a right-to-left shunt the IDC will show a biphasic humb at the ascending part of the IDC, meaning an earlier increase and decrease of the curve followed by the actual peak of a normal passage of the indicator [23, 24]. In our patients no atrial or ventricular septum defects were present. However, given the high prevalence of patent foramen ovale (PFO) of 35 % in the general population, we cannot exclude the presence of a PFO in a part of our patients [25]. Still, a PFO would probably not influence the results as only a minority of PFO’s will demonstrate a spontaneous right-to-left shunt and all patients were spontaneous breathing without respiratory distress, which means no right-to-left shunts would be expected. Indeed, most PFO’s only exhibit shunting during Valsalva manoeuvre. However, knowing that CEUS is contraindicated in patients with intra-cardiac shunts and the indicator should only pass the detection point once, the effect of an intra-cardiac shunt on the MTT of the left ventricle could be of interest for future studies [10, 26].

Our study population consisted of patients with a dilated LV and low ejection fraction; this could benefit the feasibility of ROI drawing in the LV. A larger LV with diminished contractions could facilitate ROI drawing, reducing interference with the septal and lateral walls. Indeed a higher coefficient of variation of the MTT of the much smaller, good contracting RV, 2.61 % versus 1.21 % for the LV is supported by the higher coefficient of variation of the ROI size of the RV. Nevertheless, the ICC of 0.98 for the RV is comparable with the LV ICC (Table 2). This observation suggests that contribution of ventricular size to the variability of MTT measurement is limited and implies that MTT measurement in the normal LV is as reliable.

The difference in reliability between the physicians and non-physicians could not be explained as we did not include other performance parameters in the present study, such as time to complete measurements per ROI, per patient, for the whole set of patients, and the two time moments. The explanation for this difference needs to be investigated.

Although, an ICC larger than 0.8 is still almost perfect according to Landis and Koch (1977), the intra-rater reliability per rater showed some variation with ICCs ranging from 0.81 to 0.99 (Table 4) [27]. This variation can be explained by a deviating MTT measurement at one of the time moments, which can probably be overcome by a higher number of repetitions to increase the precision [28]. In analogy to cardiac output measurements using intermittent bolus thermodilution, three repeated injections could be necessary to estimate transit times with a high precision. However, for cardiac output estimations, the use of four indicator injections improved the precision to 5 % compared to the ‘true’ value [28]. The number of repeated injections required to ensure a high precision for MTT estimation needs to be explored.

In this study, we did not investigate the effect of different ultrasonographers on the estimation of the PTT. However, we showed in an earlier study using transesophageal echocardiography that the MTT measurement is easy to perform; a good and stable view on the chamber or vessel under investigation during the whole cardiac cycle is necessary [8]. It is of importance to keep in- and expirations in a normal and regular pattern, otherwise artifacts may occur due to the displacement of the heart in the imaging window.

The development of automated algorithms for ROI definition and MTT estimation could enhance clinical feasibility of this novel CEUS tool [29]. This could create the opportunity to simplify PTT calculation at the bedside.

The relationship between the PTT and cardiac function has been investigated mainly by MRI and radionuclides [16, 30]. The relationship to different echocardiographic parameters has recently been investigated and seems promising, further investigations are necessary to evaluate its diagnostic characteristics [3].

## Conclusions

PTT assessment by drawing ROIs in CEUS recordings is a reliable technique with a high inter- and intra-rater reliability and reproducibility. Each measurement also showed a high repeatability between three different echo loops at three different doses. Differences in ROI size hardly affect the MTT per ROI. This makes this novel bedside applicable technique for measuring the PTT reliable to be performed by experienced and inexperienced operators, having an ultrasound scanner and an UCA with intravenous access. This motivates for further investigation of its clinical application in order to replace invasive measurements requiring catheterization.

## Additional file

Additional file 1:
**Apical four chamber view by contrast enhanced echocardiography.** The SonoVue® bolus passage through the right ventricle and left ventricle. ROIs were drawn in the right (red) and left ventricle (yellow) by one of the raters using Qlab®8 software. The acoustic time intensity curves are expressed in the bottom panel and both in the same color as the ROIs. (MOV 5015 kb)

## References

[1] Herold IH, Russo G, Mischi M, Houthuizen P, Saidov T, van Het Veer M (2013). Volume quantification by contrast-enhanced ultrasound: an in-vitro comparison with true volumes and thermodilution. Cardiovasc Ultrasound.

[2] Mischi M, Kalker TA, Korsten EH (2004). Contrast echocardiography for pulmonary blood volume quantification. IEEE Trans Ultrason Ferroelectr Freq Control.

[3] Brittain EL, Doss LN, Saliba L, Irani W, Byrd BF, Monahan K (2015). Feasibility and diagnostic potential of pulmonary transit time measurement by contrast echocardiography: a pilot study. Echocardiography.

[4] Choi BG, Sanai R, Yang B, Young HA, Mazhari R, Reiner JS (2014). Estimation of cardiac output and pulmonary vascular resistance by contrast echocardiography transit time measurement: a prospective pilot study. Cardiovasc Ultrasound.

[5] Ugander M, Kanski M, Engblom H, Gotberg M, Olivecrona GK, Erlinge D (2010). Pulmonary blood volume variation decreases after myocardial infarction in pigs: a quantitative and noninvasive MR imaging measure of heart failure. Radiology.

[6] Korsten HH, Mischi M, Grouls RJ, Jansen A, van Dantzig JM, Peels K (2006). Quantification in echocardiography. Semin Cardiothorac Vasc Anesth.

[7] Mischi M, Jansen AH, Korsten HH (2007). Identification of cardiovascular dilution systems by contrast ultrasound. Ultrasound Med Biol.

[8] Herold IH, Soliman Hamad MA, van Assen HC, Bouwman RA, Korsten HH, Mischi M (2015). Pulmonary blood volume measured by contrast enhanced ultrasound: a comparison with transpulmonary thermodilution. Br J Anaesth.

[9] Gorce JM, Arditi M, Schneider M (2000). Influence of bubble size distribution on the echogenicity of ultrasound contrast agents: a study of SonoVue. Invest Radiol.

[10] Senior R, Becher H, Monaghan M, Agati L, Zamorano J, Vanoverschelde JL (2009). Contrast echocardiography: evidence-based recommendations by European Association of Echocardiography. Eur J Echocardiogr.

[11] Mischi M, Kalker T, Korsten HHM (2003). Videodensitometric methods for cardiac output measurements. EURASIP J Appl Signal Processing.

[12] Sheppard CW, Savage LJ (1951). The random walk problem in relation to the physiology of circulatory mixing. Phys Rev.

[13] Kottner J, Audige L, Brorson S, Donner A, Gajewski BJ, Hrobjartsson A (2011). Guidelines for Reporting Reliability and Agreement Studies (GRRAS) were proposed. J Clin Epidemiol.

[14] Shrout PE, Fleiss JL (1979). Intraclass correlations: uses in assessing rater reliability. Psychol Bull.

[15] Bland JM, Altman DG (1986). Statistical methods for assessing agreement between two methods of clinical measurement. Lancet.

[16] Shors SM, Cotts WG, Pavlovic-Surjancev B, Francois CJ, Gheorghiade M, Finn JP (2003). Heart failure: evaluation of cardiopulmonary transit times with time-resolved MR angiography. Radiology.

[17] Streitberger A, Hocke V, Modler P (2013). Measurement of pulmonary transit time in healthy cats by use of ultrasound contrast media “Sonovue(R)”: feasibility, reproducibility, and values in 42 cats. J Vet Cardiol.

[18] Wise ME (1966). Tracer dilution curves in cardiology and random walk and lognormal distributions. Acta Physiol Pharmacol Neerl.

[19] Piscaglia F, Bolondi L (2006). The safety of Sonovue in abdominal applications: retrospective analysis of 23188 investigations. Ultrasound Med Biol.

[20] Rognin NG, Frinking P, Costa M, Arditi M. In-vivo perfusion quantification by contrast ultrasound: Validation of the use of linearized video data vs. raw RF data. Ultrasonics Symposium, 2008 IUS Proceedings, IEEE 2008:1690-3.

[21] Gauthier TP, Averkiou MA, Leen EL (2011). Perfusion quantification using dynamic contrast-enhanced ultrasound: the impact of dynamic range and gain on time-intensity curves. Ultrasonics.

[22] Cao JJ, Wang Y, McLaughlin J, Haag E, Rhee P, Passick M (2011). Left ventricular filling pressure assessment using left atrial transit time by cardiac magnetic resonance imaging. Circ Cardiovasc Imaging.

[23] Giraud R, Siegenthaler N, Park C, Beutler S, Bendjelid K (2010). Transpulmonary thermodilution curves for detection of shunt. Intensive Care Med.

[24] Michard F, Alaya S, Medkour F (2004). Monitoring right-to-left intracardiac shunt in acute respiratory distress syndrome. Crit Care Med.

[25] Meier B, Lock JE (2003). Contemporary management of patent foramen ovale. Circulation.

[26] Zierler KL (1962). Theoretical basis of indicator-dilution methods for measuring flow and volume. Circ Res.

[27] Landis JR, Koch GG (1977). The measurement of observer agreement for categorical data. Biometrics.

[28] Nilsson LB, Nilsson JC, Skovgaard LT, Berthelsen PG (2004). Thermodilution cardiac output--are three injections enough?. Acta Anaesthesiol Scand.

[29] Saporito S, Herold IH, Houthuizen P, van den Bosch HC, Korsten HH, van Assen HC (2015). Automatic indicator dilution curve extraction in dynamic-contrast enhanced imaging using spectral clustering. Phys Med Biol.

[30] Jones RH, Sabiston DC, Bates BB, Morris JJ, Anderson PA, Goodrich JK (1972). Quantitative radionuclide angiocardiography for determination of chamber to chamber cardiac transit times. Am J Cardiol.

